# Genetic lesions in nodular lymphocyte-predominant Hodgkin lymphoma and T cell/histiocyte-rich large B-cell lymphoma identified by whole genome sequencing

**DOI:** 10.1038/s41375-025-02679-3

**Published:** 2025-07-16

**Authors:** Tobias Rausch, Hendrik Schäfer, Julia Bein, Felix Mölder, Lilian Kara Beeck, Bernd Kölsch, Sabrina Borchert, Vladimir Benes, Teresa Halbsguth, Uta Brunnberg, Thomas Oellerich, Thomas Tousseyn, Maurilio Ponzoni, Johannes Köster, Martin-Leo Hansmann, Ralf Küppers, Sylvia Hartmann

**Affiliations:** 1 GeneCore, European Molecular Biology Laboratory, Heidelberg, Germany; 2 Institute of Pathology, University Hospital Essen, University Duisburg-Essen, Essen, Germany; 3 Dr. Senckenberg Institute of Pathology, Goethe University Frankfurt am Main, Theodor-Stern-Kai 7, Frankfurt am Main, Germany; 4 Institute of Cell Biology (Cancer Research), Faculty of Medicine, University of Duisburg-Essen, Essen, Germany; 5 Department of Internal Medicine 2, Hospital of the Goethe University, Frankfurt am Main, Germany; 6 Frankfurt Cancer Institute, Goethe University, Frankfurt am Main, Germany; 7 University Cancer Center (UCT) Frankfurt, University Hospital, Goethe University, Frankfurt am Main, Germany; 8 German Cancer Consortium (DKTK) partner site Frankfurt/Mainz, Frankfurt am Main, Germany; 9 Department of Pathology, University Hospitals K.U. Leuven, Leuven, Belgium; 10 Unit of Lymphoid Malignancies, Department of Pathology, Scientific Institute San Raffaele, Milan, Italy; 11 Bioinformatics and Computational Oncology, Institute for Artificial Intelligence in Medicine, University Hospital Essen, Essen, Germany; 12 Frankfurt Institute for Advanced Studies, Frankfurt am Main, Germany; 13 Institute of General Pharmacology and Toxicology, Goethe University Frankfurt am Main, Frankfurt am Main, Germany; 14 German Cancer Consortium (DKTK) partner site, Essen, Germany

**Keywords:** Cancer genetics, Oncogenesis

## Abstract

Nodular lymphocyte-predominant Hodgkin lymphoma (NLPHL) is a rare malignant lymphoma characterised by a few large tumour cells expressing B-cell antigens in an inflammatory background. T-cell/histiocyte-rich large B-cell lymphoma (THRLBCL) is now considered to be closely related to NLPHL. Little is known about the mutational spectrum of the lymphoma cells in primary NLPHL and THRLBCL due to the rarity of the diseases and the technical challenges of analysing these tumours. Therefore, the aim of the present study was to elucidate mechanisms contributing to the pathogenesis of NLPHL and THRLBCL by whole genome sequencing of laser microdissected tumour cells from seven cases. We observed a heterogeneity of transforming events, with cases showing abundant somatic mutations, others with a predominance of structural variations, and cases with few aberrations. The genes that were most frequently affected by aberrations encode factors influencing JAK-STAT, NF-κB, and/or WNT signaling, and apoptosis regulators. However, the mutated genes, such as *SOCS3, JUNB, IRF1* and *ITPKB*, were not the typical targets known from classical Hodgkin lymphoma (cHL). Two cases showed recurrent rearrangements of *BCL6* and *CD74*. In conclusion, our data enrich our understanding of NLPHL and THRLBCL and highlight common and distinct features with respect to cHL.

## Introduction

Nodular lymphocyte-predominant Hodgkin lymphoma (NLPHL), also called nodular lymphocyte-predominant B-cell lymphoma [1, 2], is a unique type of malignant lymphoma that shares features with both classic Hodgkin lymphoma (cHL) and other B-cell lymphomas. NLPHL mainly affects young adults [3] with a male predominance among patients of about 3:1 [4]. It often presents with slowly growing, enlarged, sometimes bulky lymph nodes. Most patients present with early stage disease and have excellent outcomes with stage-adapted therapies [5, 6]. However, different histopathological growth patterns have been identified [7]. Variant growth patterns, particularly pattern D and E according to Fan et al. [7], are more common in patients with advanced stage disease [8–11]. Patients with variant growth patterns have a higher risk of relapse in the first five years after diagnosis [8] and a higher risk of transformation [12]. NLPHL patients with relapse or progression within the first 24 months after diagnosis have a dramatically reduced overall survival [13, 14]. There is a particular need to learn more about the pathogenesis of these high-risk NLPHL to improve the management of these patients.

T-cell/histiocyte rich large B-cell lymphoma (THRLBCL) is a lymphoma with many features in common with NLPHL variants, particularly pattern E. It also shows a male predominance, a comparable immunophenotype of the tumor cells [15] and usually an advanced clinical stage [16]. Therefore, NLPHL variant patterns and THRLBCL have recently been considered to be a spectrum of the same disease [15, 17].

Previously, it was observed that LP cells, the neoplastic cells of NLPHL, often have diploid nuclei and can harbor a variable amount of genomic gains and losses [18]. In this first study using classical comparative genomic hybridization (CGH) in NLPHL, the overall number of aberrations was higher when compared with THRLBCL [19]. Using array CGH, we previously observed an inverse trend and also common aberrations between NLPHL and THRLBCL [20]. Mutational screening has hitherto been confined to transformations of NLPHL, due to the low tumor cell content in the tissue and the immunophenotype of the LP cells, which is highly similar to that of germinal center B cells. This has rendered the purification of isolated tumor cells at high numbers impossible. PCR analysis of isolated LP cells identified recurrent *SOCS1* mutations [21], and whole-genome sequencing of two cases of transformed NLPHL led to the identification of frequent mutations in *JUNB*, *CREBBP*, *DUSP2* and *SGK1* [22], which were also found to be mutated in THRLBCL [23]. Whole exome sequencing of aggressive B-cell lymphomas identified a cluster called “ST2”, putatively transformed from NLPHL and harboring truncating mutations in *TET2* and *SGK1* and inactivating mutations in *DUSP2*, *SOCS1* and *NFKBIA*. Another study of transformed NLPHL by Song et al. [24]. confirmed frequent mutations in *CARD11*, *JUNB*, *BCL10*, *NFKBIA*, *NOTCH2*, *EP300*, *CREBBP*, *TNFAIP3* and *TET2*. Whole-genome sequencing data from isolated tumor cells from primary NLPHL cases are still lacking. The aim of the present study was therefore to learn more about the genetics of primary NLPHL, especially those cases with a variant growth pattern E/THRLBCL and a high medical need.

## Methods

### Patients and laser microdissection

Samples from patients with NLPHL and THRLBCL were selected based on the availability of frozen tissue with good morphology, relative abundance of LP cells and a prevalence of atypical histological growth patterns. The local ethics committee of Goethe University Hospital approved the study (SHN-6-2018) and informed consent from all patients was obtained in accordance with the Declaration of Helsinki. Details on the clinical data of the patients are provided in Table 1. For purification of the tumor cells, 3000 single LP tumor cells were laser microdissected per case as previously described applying OCT2-immunostaining [20]. For comparison, 3000 reactive, OCT2-negative lymphocytes from the lymphoma microenvironment were separately microdissected. DNA was extracted using an ethanol precipitation complemented with glycogen.

**Table 1:** Clinical data on patients.

Case ID	Histopatho-logical growth patterns	Gender	Age at diagnosis (years)	Outcome	Stage at diagnosis	First manifestation or relapse	B-cell receptor reactivity of LP cells according to refs. [63, 64]
1	NLPHL patterns C/E	m	51	D	I	Relapse	No antigen identified
2	NLPHL pattern E	m	43	CR	IVB	First manifestation	Not done
3	NLPHL pattern E	m	44	DOD	IVB	Relapse	*R. mucilaginosa*
4	NLPHL patterns C/E	m	26	Primary refractory, CR^a^	II	First manifestation	*M. catarrhalis*
5	THRLBCL	m	27	DOD	IVB	First manifestation	Not done
6	THRLBCL	m	20	DOD	IVB	First manifestation	Not done
7	NLPHL pattern E	m	50	DOD	IVB	First manifestation	Not done

*D* death, *DOD* death of disease, *CR* complete remission.

^a^The patient was first refractory, then achieved complete remission after salvage therapy.

### Library preparation and whole-genome sequencing

For library preparation, the NEBNext Ultra II Kit (New England Biolabs, Ipswich, MA, USA) was used with 9–18 cycles of amplification depending on the DNA amount obtained. Libraries were sequenced on a HiSeq X Ten machine at the Deutsches Krebsforschungszentrum (DKFZ, Heidelberg).

### Genome alignment and variant calling

Sequenced reads were aligned to the GRCh38 human reference genome using bwa and alignments were sorted and indexed using samtools [25]. Alignment quality control was performed using samtools and alfred [25]. Mutect2 (10.1101/861054) was employed to call and genotype short variants (single-nucleotide variants and short insertions and deletions), which were subsequently normalized and left-aligned using bcftools [25]. VEP [26] was used to annotate all short variants for biological consequences. Short variants were phased using ShapeIt4 with the haplotype reference panel of the expanded 1000 Genomes Project cohort [27, 28]. To minimise the number of false positives, at least 2 reads supporting the somatic variant and a minimum variant allele frequency ≥0.05 were required.

Larger structural and copy-number variants were called using delly [29] and Manta [30]. The read-depth signal was segmented using the DNAcopy Bioconductor package [31]. Copy-number variants and structural variants were visualized using wally [32] and custom R scripts using ggplot2. The scripts used in this study are openly published in a GitHub repository under the MIT License (10.5281/zenodo.10417638). Tumor purity and ploidy were estimated using PURPLE [33]. For determination of the mutational signatures the Bioconductor package MutationalPatterns was used [34].

The quality of the sequencing results of cases 1–3 and 5–7 was satisfactory. In case 4 contaminating vector sequences mapping exclusively to the exonic regions of ERBB2, CD30 and FOXO1 were identified. As repetition of this sample failed several times, the loci affected by the contaminating sequences were excluded from further analysis and the case was excluded from the overall comparison of structural variants.

The microbial content of the whole-genome sequencing data of all samples was analyzed using Kraken2 [35] using the so-called standard database of kraken2 to classify human as well as bacterial reads. Using this database, we computed absolute and relative read counts (normalized to overall bacterial content) for Rothia mucilaginosa and Moraxella catarrhalis.

### Immunofluorescence double stainings

Immunofluorescence staining was performed as previously described [36]. Antibodies to BCL6 (clone m7211, Agilent-DAKO, Santa Clara, USA) and CD74 (HPA-010592, Atlas antibodies, Stockholm, Sweden) were applied at dilutions of 1:20 and 1:250, respectively. The Vectafluor Duet Double labeling kit (Vector, Newark, CA, USA) was used for detection.

## Results

### Patient characteristics

All seven patients were male. The mean age was 43 years (range 20–51 years, Table 1). All patients had either high-risk NLPHL or THRLBCL. Two patients had pure THRLBCL and three patients had a THRLBCL-like NLPHL variant pattern E according to Fan et al. [7] (Supplementary Fig. 1). Two patients had areas of growth pattern C according to Fan et al. with smaller areas of pattern E. Thus, all patients had NLPHL variants or THRLBCL, that is related to NLPHL. Four out of five patients with stage IVB died of disease. One patient with stage IVB and NLPHL growth pattern E achieved complete remission after BEACOPP-based therapy. In general, the patients studied presented NLPHL/THRLBCL tumors with a high risk profile (Table 1).

### NLPHL and THRLBCL often show diploid tumor cells and heterogeneous activity of mutational processes

**Fig. 1:**
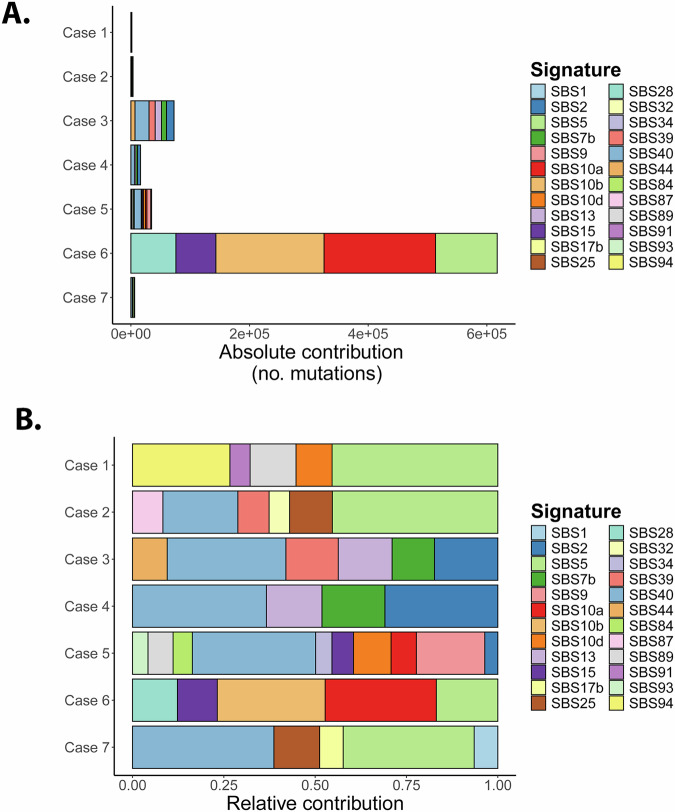
Distribution of cosmic mutation signatures in absolute and relative frequencies in the seven cases of NLPHL/THRLBCL. **A** Absolute distribution of cosmic signatures. **B** Relative distribution of cosmic signatures. SBS single base substitution.

The mean coverage of the tumor samples was 73.4x and after removal of duplicates 44.6× (Supplementary Table 1). For SNV analysis, all variants with allele frequencies ≥5% were considered. Ploidy levels of all tumors were calculated and were found to be diploid in four out of the seven samples. Cases 3, 6 and 7 had tetraploid tumor cells consistent with whole-genome duplication, followed by subsequent chromosomal alterations (Supplementary Fig. 2). Mutational signatures were calculated for all cases based on the base triplet exchanges according to Alexandrov [37]. No predominant signature was identified that was common to all samples (Fig. 1). Case 6, which had the highest number of SNVs of all cases, showed an important influence of mutation signatures SBS10a and 10b (30% each). Both signatures correspond to DNA polymerase epsilon exonuclease domain mutations and both tend to generate large numbers of somatic mutations (>100 mutations per MB), so samples with these signatures were termed hypermutators. Case 6 indeed presented the mutation p.Arg1390Cys in *POLE*, deleterious according to SIFT. Case 5 showed little activity of signature SBS10a (7%). None of the other cases showed activity of SBS10a or b. In contrast, cases 2, 3, 4, 5 and 7 had a relatively high activity of SBS40 (21-39% of mutations), a signature of unknown etiology. Another prominent signature of unknown etiology with clock-like feature was SBS5, present in cases 1, 2, 6 and 7 (17–45% of mutations). SBS2 was observed in cases 3, 4 and 5 (17, 31 and 4%, respectively), related to AID/APOBEC activity as is SBS13, which was also present in cases 3 and 4 (15% each). The greatest similarity between their mutation signatures was found in cases 3 and 4. SBS9, which is related to somatic hypermutation, was present in case 5 (19%).

### Mechanisms of lymphomagenesis are heterogeneous in NLPHL

The seven cases analyzed showed a large variance in the number of single nucleotide variants (SNV) and structural variations (SV, Table 2). Case 2, with a clinical complete response, had the lowest number of aberrations overall with 26 coding SNV and three breakpoints. In contrast, case 6 had the highest number of pathogenic SNVs (*n* = 4527). The genomic aberrations in case 3 were dominated by non-synonymous SNVs (*n* = 843) and deletions (*n* = 491), and multiple genomic breakends were also observed (*n* = 24). Therefore, the mechanisms of lymphomagenesis in NLPHL and THRLBCL are heterogeneous.

**Table 2:** Numbers of genetic aberrations observed per case.

Case ID	Histopathological growth patterns	Nonsynonymous somatic SNV & indels	DEL	INV	DUP	BND
1	NLPHL C/E	263	0	5	0	12
2	NLPHL pattern E	26	0	0	0	3
3	NLPHL pattern E	843	491	32	43	24
4	NLPHL C/E	209	96	193	13	Not applicable^a^
5	THRLBCL	247	49	11	2	8
6	THRLBCL	4527	30	13	5	46
7	NLPHL pattern E	58	5	8	2	4

*SNV* single nucleotide variants, *DEL* deletions, *INV* inversions, *DUP* duplications, *BND* break-ends.

^a^Due to the presence of contaminating vector sequences, genome-wide calling of break-ends was not reliable for case 4.

### Mutations in NLPHL are associated with JAK-STAT signaling, WNT signaling, AP1 transcription factor and homologous recombination deficiency (HRD)

**Fig. 2:**
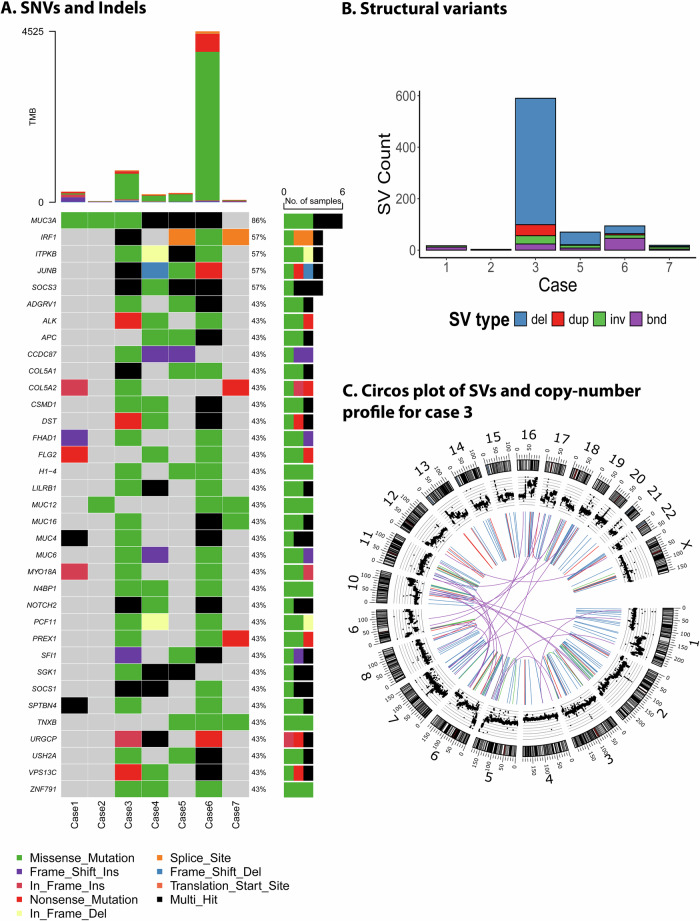
Somatic SNV and InDel mutations and genomic aberrations in the seven cases of NLPHL/THRLBCL. **A** Non-synonymous single nucleotide variants (SNVs) and Indels and distribution of SNV types (missense mutations, stop-gain mutations, other mutations including splice site mutations and frameshift mutations) listed by frequency (number of cases, number of variants per case). **B** Structural variants (SVs) per case with del: deletion-type rearrangements, dup: duplication-type rearrangements, inv: inversion-type rearrangements and bnd: inter-chromosomal rearrangements. Case 4 was excluded from this figure due to the contaminating vector sequences. **C** Circos plot of SVs and copy-number profile for case 3. From outside to inside, the tracks are: chromosome ideogram, read-depth, large (>10 Kbp) SVs, and inter-chromosomal rearrangements. Color scale is the same as in (**B**).

The genes most frequently affected by somatic mutations were *MUC3A, IRF1*, *ITPKB, JUNB and SOCS3* (each mutated in ≥4 cases). Among the genes mutated in at least three cases, were previously described genes like *SOCS1* and *SGK1* (Fig. 2A).

In addition to various MUC genes, *ADGRV1, ALK, APC, CCDC87, COL5A1, COL5A2, CSMD1, DST, FHAD1, FLG2, H1-4, LILRB1*, involved in regulation of immune responses, *MYO18A, NOTCH2, N4BP1, PCDF11, PREX1, SFI1, SPTBN4, TNXB, URGCP, USH2A, VPS13C* and *ZNF791* were each mutated in 3 cases (43%).

*MUC3A* had eleven missense variants in cases 1, 3, 4, 5, and 6 and one stop gain in case 6. *JUNB*, an AP-1 transcription factor subunit, showed five frameshift and stop variants in cases 3, 4 and 6 and one missense variant in case 5. *SOCS3* showed eight missense variants and one start loss. *ITPKB* had a stop gain and frameshift deletion in case 5 and four missense variants in cases 3, 4, 5 and 6 and an inframe deletion and additional structural variants (deletions) of exons 3–8 in case 4. *IRF1* presented three missense variants and two splice variants in cases 3, 5, 6 and 7. *SGK1* showed one stop codon, seven missense and one splice variants in cases 3, 4 and 5. Case 3, which had a missense mutation in *SGK1*, also had a deletion of exon 1 of *SGK1*. The *APC* gene showed a stop-gain variant in case 6 and missense variants in cases 4 and 5. In addition, a stop-gain of *APC2*, an APC-related gene also involved in WNT signaling, was found in case 3 and a missense variant of *APC2* in case 6.

Notably, numerous of the recurrent somatic mutations affected members of the signaling pathways JAK/STAT (*SOCS1, SOCS3*), AP1 (*JUNB*), and WNT (*APC, APC2*).

The presence of SNVs in HRD-related genes was also analyzed. The four cases 3, 4, 5 and 6 had one or more HRD-related genes affected by SNVs (Table 3). Case 6, which had the highest number of SNVs, had missense mutations in *ATM, POLE* and *BRCA2*, as well as known pathogenic mutations from ClinVar in MSH6, RAD50, APC and NF1, likely explaining the high mutational load in this tumor.

**Table 3:** Mutations identified in LP tumor cells in HRD-related genes.

Gene	Case 1	Case 3	Case 4	Case 5	Case 6
ATM					p.Ser1250Tyr *
BARD1		p.Asp172His *	p.Ser186Gly *		
BLM		p.Leu1042TrpfsTer2 +			
BRCA1				p.Asp1546Asn *	p.Lys1207Asn *
BRCA2	p.Asp381Tyr *				p.Ala1648Thr *
BRIP1		p.Met425Val *			p.Val894Ala, p.Arg162Ter #
NBN		p.Gln448Ter #			p.Glu383Ter #
RAD50					p.Glu1033Ter #

Mutation consequence is indicated : * missense mutation, # stop-gain, + frameshift. In cases 2 and 7, no mutations in homologous repair deficiency (HRD)-related genes were observed.

We also searched for constitutional variants that were present in the lymphoma and non-tumor cells of a given case, focusing on events with known pathogenetic relevance. This revealed constitutional *FAS* variants, as previously described [38], in cases 2 (missense) and 5 (splice donor variant).

Since an association of NLPHL with *Rothia mucilaginosa* and *Moraxella catarrhalis* was recently described [39, 40], sequencing data were screened for the presence of bacterial reads. 5 tumors (Case 1, 3, 4, 5, and 7) and 1 control sample (Case 4) had more than 1000 reads assigned to Rothia mucilaginosa suggesting the presence of R. mucilaginosa in these samples. However, the relative numbers were all below 1% and a contamination can therefore not be excluded (Supplementary Table 2).

### Copy number changes of limited size affect genes involved in JAK-STAT signaling, immune escape and apoptosis inhibition

**Fig. 3:**
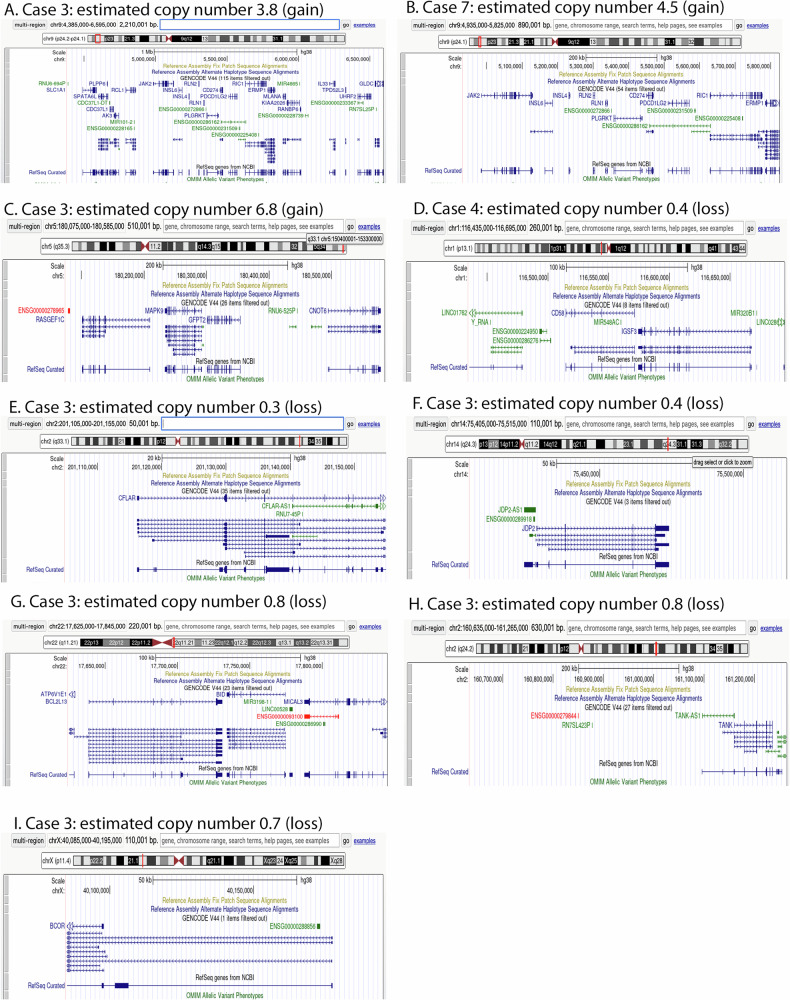
Copy number aberrations of limited size covering individual or few genes in individual cases. Copy number (CN) gains are shown in panel A, B, and C. Copy-number losses in D, E, F, G, H, and I. The genes located in the specific region are indicated along with the case copy number (balanced copy number is 2). Since the microdissected tumor cells were not 100% pure, we assume that a copy number of 0.3 is likely to be a homozygous loss.

Abundant copy number aberrations were observed. Some affected loci contained only one or few genes, potentially limiting the effect of the observed genomic aberration to a specific gene (Fig. 3). These included copy number (CN) gains of chromosome 9p affecting *JAK2, PD-L1* and *PD-L2* in two cases (estimated CN of 4.5 for chr9:4,935,000–5,825,000 in case 7, estimated CN of 3.8 for chr9:4,385,000–6,595,000 in case 3, Fig. 3A, B, balanced CN is 2), and another gain (CN: 6.8) of chr5:180,075,000–180,585,000 including *MAPK9* in case 3 (Fig. 3C). Case 4 showed a presumed homozygous loss of chr1:116,435,000–116,695,000 (CN 0.3) including the two genes *CD58* and *IGSF3* (Fig. 3D). Case 3 presented multiple localized presumed homozygous losses of the regions chr2:201,105,000–201,155,000 including the transcription start sites and first exons of *CFLAR* (CN 0.3), chr14:75,405,000–75,515,000 including the entire sequence of *JDP2* (CN 0.4, Fig. 3E, F), another component of the AP-1 transcription factor. In addition, heterozygous (or subclonal homozygous) losses were observed in this case, including *BCL2L13* and *BID*, encoding pro-apoptotic factors of the BCL2 family (chr22:17,625,000–17,845,000, CN 0.8, Fig. 3G) and *TANK* (chr2:160,635,000–161,265,000, CN 0.8, Fig. 3H), a TRAF family member and negative regulator of NF-κB, which additionally had a breakend and an inversion-type rearrangement in case 4. Case 3 also presented a probably subclonal deletion of the *BCOR* gene on chrX:40,085,000–40,195,000, CN 0.6, Fig. 3I).

### NLPHL cases show a variety of structural variants, including recurrent rearrangements of *BCL6* and *CD74* in two cases

A variety of structural variants were found in the seven NLPHL cases (Fig. 2B). Structural variants including copy number aberrations and translocations were additionally detected by a different algorithm than SNVs and indels (delly [29] and manta [30]).

Case 3 showed the largest number of somatic structural variants and a highly altered copy number landscape (Fig. 2C) with several chromosomes exhibiting oscillating copy numbers, suggesting a chromothripsis SV formation process.

*WWOX*, a tumor suppressor that plays a role in apoptosis and inhibits WNT signaling, had the highest number of structural variants, with 7 variants in three cases.

**Fig. 4:**
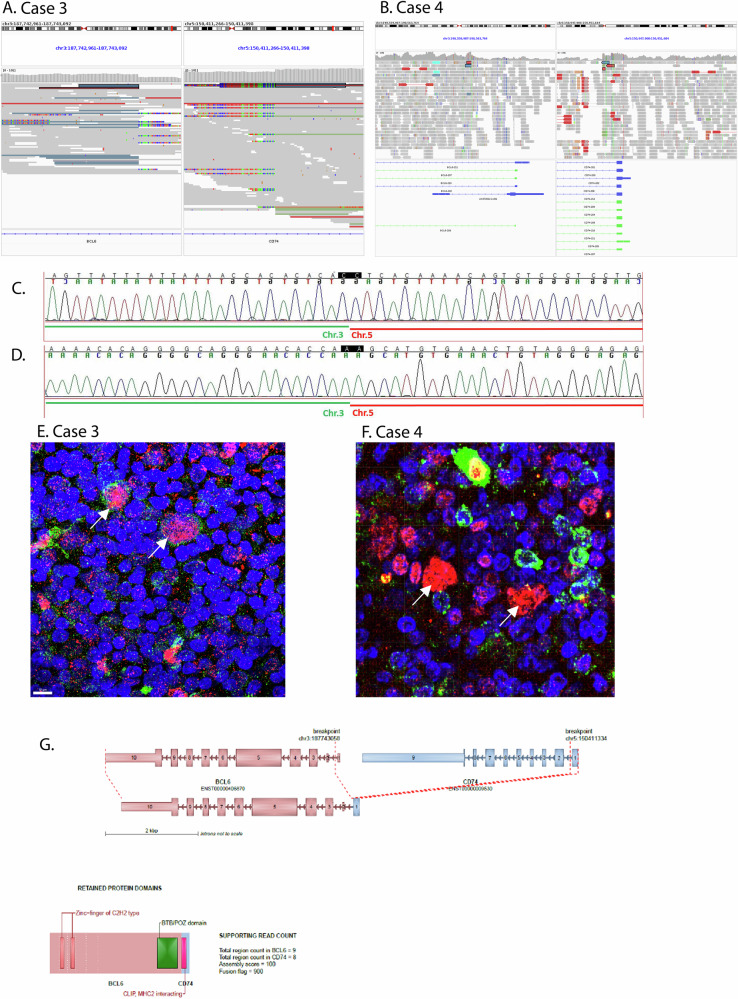
*BCL6*-*CD74* rearrangements in cases 3 and 4. **A**, **B** Overview of the observed breakpoints in case 3 and case 4 using IGV [68] plots. **C** Breakpoint-spanning Sanger sequence obtained from the rearrangement in case 3. **D** Breakpoint-spanning Sanger sequence obtained from the rearrangement in case 4. **E** Immunofluorescence double staining of BCL6 (red) and CD74 (green) with DAPI counterstaining showing strong nuclear expression of BCL6 in the LP cells of case 3 and weak membrane bound expression of CD74 (arrows). **F** Immunofluorescence double staining of BCL6 (red) and CD74 (green) with DAPI counterstaining showing strong nuclear expression of BCL6 in the LP cells of case 4 with absence of CD74 expression (arrows). Preserved membrane bound expression of CD74 is observed in non-tumor cells. **G** Schematic representation of rearrangement in case 3 using BrassVis [69].

In cases 3 and 4, a t(3;5) translocation was identified involving the genes *BCL6* and *CD74* (case 3: chr3:187,743,058; chr5:150,411,334; case 4: chr3:187,744,493; chr5:150,412,329, Fig. 4). In both cases, the breakpoints were in intron 1 of *BCL6* and also intron 1 of *CD74*. At the protein level, LP cells of both cases showed nuclear expression of BCL6 protein (Fig. 4). However, membrane bound expression of CD74 was only weakly detected in case 3, whereas CD74 protein was absent in the LP cells of case 4 (Fig. 4). We tested 13 further NLPHL cases for CD74 protein expression by immunohistochemistry and all cases had CD74-positive LP cells (data not shown).

### Comparison with SNVs in cHL and diffuse large B-cell lymphoma (DLBCL)

**Fig. 5:**
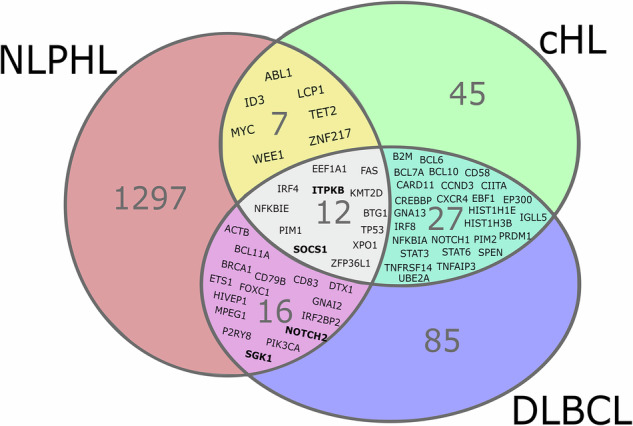
Mutational overlap with cHL and DLBCL. Considered were all nonsynonymous SNV present in at least one case in the present study (excluding the hypermutated case 6) and/or recurrently mutated in at least one of the publications on 639 cHL patients [38–42] and/or 878 DLBCL patients [43, 44]. Genes that were affected by nonsynonymous SNVs in more than one case in the present study are printed in bold.

The newly obtained data from seven NLPHL/THRLBCL were also compared with SNVs found by whole exome sequencing and targeted sequencing in 639 cHL patients [41–45] and 878 DLBCL patients [46, 47]. However, the techniques used are different, as cHL requires either the purification of tumor cells from tissue or a liquid biopsy-based approach. Due to the small number of cases in the present study, only limited conclusions can be drawn from this comparison. When comparing all SNVs that were found in our study, excluding the hypermutated case 6, there was an overlap with cHL with 7 mutated genes, with DLBCL with 16 mutated genes and with all three categories with 12 mutated genes (Fig. 5). It is noteworthy that JAK/STAT pathway mutations, aberrations in *ITPKB*, *BCOR*, *CD58* as well as copy number gains *PD-L1* and *PD-L2*, have been found recurrently in cHL as well as in NLPHL. Conversely, other recurrently mutated genes in NLPHL, such as *JUNB*, *SOCS3* and *IRF1*, have been found to occur at a significantly lower frequency in cHL and DLBCL.

## Discussion

Although cHL has been characterized by several next-generation sequencing studies [41–45, 48], to our knowledge there are so far no such studies for NLPHL or THRLBCL. This is partly due to the fact that NLPHL is less common than cHL. On the other hand, recent studies in cHL have derived genomic data from cell-free DNA from plasma [43, 45, 48], a technique that has been successfully established in cHL, where the neoplastic cells are pre-apoptotic and shed abundant DNA variants into the plasma. This has not yet been demonstrated in tumors from the NLPHL/THRLBCL spectrum. Therefore, to the best of our knowledge, we present here the first whole-genome sequencing data obtained from isolated tumor cells of primary NLPHL and THRLBCL.

We found a heterogeneity in the mechanisms leading to lymphomagenesis with case 3 harboring a high number of deletions and case 6 numerous SNVs, whereas cases 2 and 7 had a relatively low number of aberrations overall. Case 6 also had a high number of mutations in HRD-related genes, as did cases 3–5 to some extent. The present study confirms previously described genes mutated in NLPHL and transformed NLPHL such as *SOCS1* [21], *SGK1* and *JUNB* as well as copy number gains of chromosome 9p [24, 49]. However, we extend our knowledge of mutations for some novel candidates as we also observed other members of the JAK-STAT-signaling pathway like *SOCS3* to be affected by mutations (4/7 cases, 57%). *SOCS3* was shown to promote apoptosis and downregulate NF-κB activity when SOCS3 was reconstituted in SOCS3-negative mantle cell lymphoma [50]. *IRF1*, also mutated in 4/7 cases (57%), both suppresses tumor cell growth and stimulates an immune response against tumor cells [51]. *ITPKB*, mutated in 4/7 cases (57%), is also recurrently mutated in cHL [44, 48, 52]. Deficiency of ITPKB was found to be associated with common variable immunodeficiency [53, 54].

*N4BP1*, which had three missense variants, is a potent suppressor of cytokine production that acts as a negative regulator of Toll-like receptor (TLR)-induced cytokine and chemokine responses, thereby limiting inflammatory cytokine responses to minor insults. N4BP1 inhibits TLR-dependent activation of NF-κB by interacting with the NF-κB signaling essential modulator (NEMO) [55]. Inactivation of *N4BP1* may therefore contribute to both NF-κB activity in LP cells and the composition of the prominent inflammatory microenvironment in NLPHL. The high number of mutations in MUC genes may be an artifact related to the large size of the genes [56].

Several apoptosis regulators such as *BID, BCL2L13, WWOX* and *FAS* were affected by aberrations, suggesting that the LP cells are rescued from apoptosis by inactivation of proapoptotic factors as was seen in Hodgkin-Reed-Sternberg cells in cHL [57].

Translocations affecting the *BCL6* locus have been previously described in NLPHL [58–61], often with an immunoglobulin family member as translocation partner. The novel finding here is the identification of *CD74* as a recurrent translocation partner to *BCL6* in two cases, that had either *M. catarrhalis*- or *R. mucilaginosa*-reactive B-cell receptors. We could detect only weak CD74 protein expression in the LP cells of case 3 and loss of CD74 protein expression in case 4, suggesting that the main effect of this translocation may be the localization of BCL6 in proximity to the active promoter of CD74, finally leading to independency from antigenic stimulation. Both cases with *BCL6*-*CD74* rearrangement had bacteria-reactive B-cell receptors of the LP cells and they were among the cases with a high mutation load attributed to similar mutational signatures. The high activity of SBS13 (related to AID/APOBEC) in cases 3 and 4 suggests the hypothesis that the *BCL6-CD74* rearrangement could be an off-target effect of AID somatic hypermutation. Consequently, the prevailing hypothesis is that the bacterial infection is the initiating factor for tumor genesis. In line with prior reports [62, 63], CD74 was expressed in the LP cells of 13 non-related NLPHL cases, and also in the NLPHL cell line DEV [60]. CD74 is the invariant chain of MHC class II, plays a critical role in MHC class II antigen processing [64] and is the cell surface receptor for the cytokine macrophage migration inhibitory factor (MIF), which, when bound to the encoded protein, activates NF-κB signaling and leads to increased cell survival [65, 66]. Translocations involving *CD74* have also been observed in lung cancer [67]. However, in lung cancer expression of fusion proteins could be demonstrated, which we could not demonstrate in the two NLPHL cases.

The conclusion of the comparison of genes mutated in cHL and DLBCL is limited, as the methods and the case numbers differ between the studies. Despite the demonstration of shared genetic alterations with cHL (e.g., *SOCS1, PDL1, PDL2, BCOR, CD58*), NLPHL presents a range of distinct mutations that regulate JAK-STAT activity and immune escape in LP cells through alternative mechanisms compared to those observed in cHL.

In summary, this study builds on our understanding of genomic variants in NLPHL and THRLBCL, highlighting both similarities and differences with cHL and for the first time describing rearrangements of *BCL6* involving *CD74* in NLPHL.

## Supplementary information

Supplementary Figures and Tables

## Data Availability

Raw data are available from the corresponding author on reasonable request.
