# Supplementary material for: Genetic lesions in nodular lymphocyte-predominant Hodgkin lymphoma and T cell/histiocyte-rich large B-cell lymphoma identified by whole genome sequencing
Source: Leukemia. 2025 Jul 16;39(9):2215–25. doi: 10.1038/s41375-025-02679-3 (PMC12380620; doi:10.1038/s41375-025-02679-3)
Supplement: Supplementary file 1 — Supplementary Figures and Tables [file 41375_2025_2679_MOESM1_ESM.docx]

**Supplementary Figures**


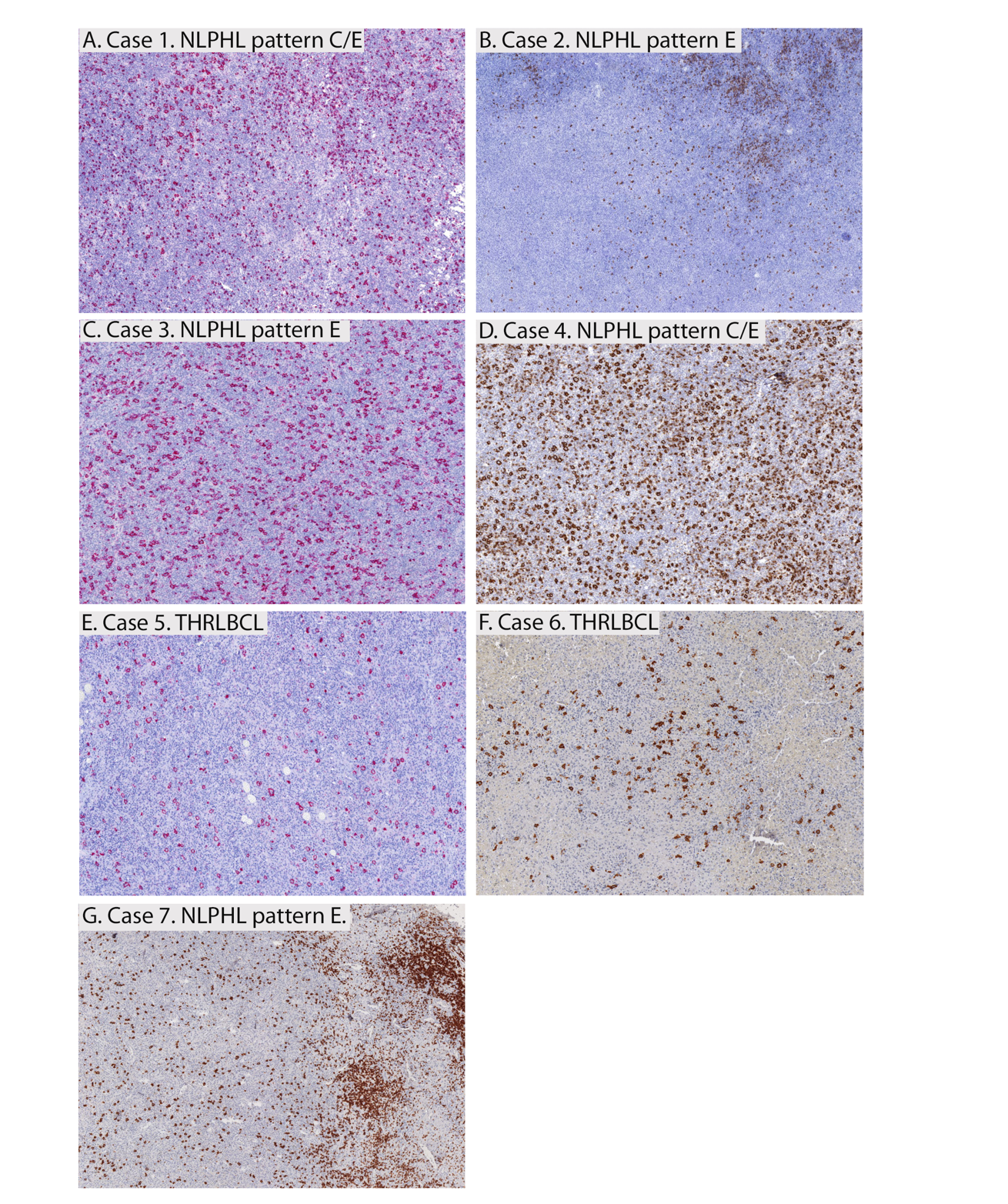


**Suppl. Figure 1. Representative histologic images from the seven cases studied by whole genome sequencing.**

All images represent CD20-immunostainings with either red or brown detection system.

A. Case 1, 6-fold magnification, B. Case 2, 3-fold magnification, C. Case 3, 6-fold magnification, D. Case 4, 6-fold magnification, E. Case 5, 6-fold magnification, F. Case 6, 8-fold magnification, G. Case 7, 5-fold magnification.


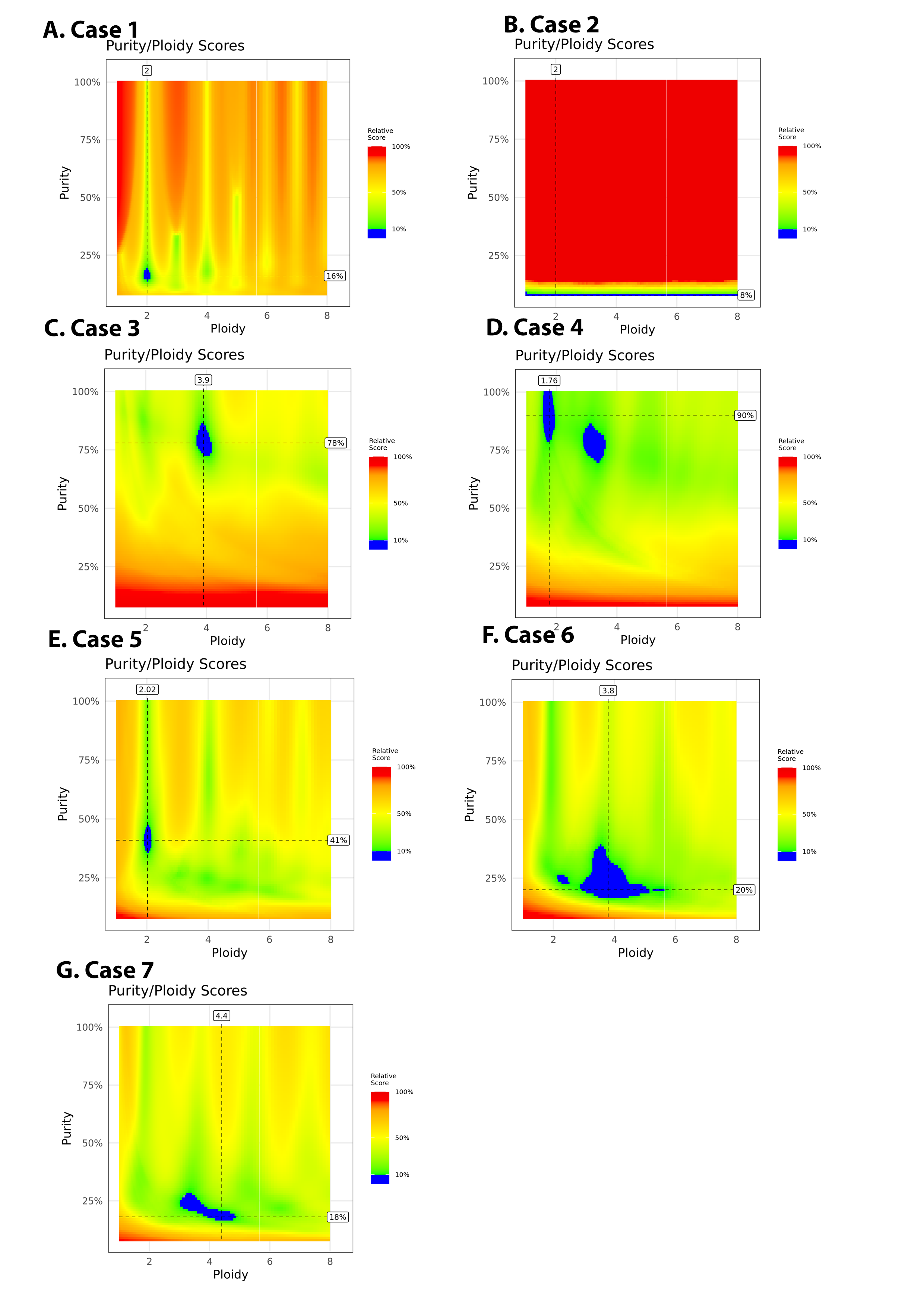


**Suppl. Figure 2. Purity of tumour samples and ploidy levels in all 7 cases determined according to the distribution of variant allele frequencies in WGS.**

**Suppl. Tables**

**Suppl. Table 1.** Coverages obtained by sequencing normal and tumour DNA. The objective was to achieve a coverage of 60x for the tumour sample and 30x for the normal DNA sample. Raw coverage includes all obtained reads, unique coverage is the coverage after removal of duplicates.

| **Case** | **Raw coverage Normal (x)** | **Unique coverage Normal (x)** | **Raw coverage Tumor (x)** | **Unique coverage Tumor (x)** |
| --- | --- | --- | --- | --- |
| **1** | 38 | 36 | 64 | 36 |
| **2** | 42 | 22 | 86 | 58 |
| **3** | 41 | 36 | 76 | 55 |
| **4** | 35 | 15 | 61 | 15 |
| **5** | 36 | 22 | 78 | 56 |
| **6** | 39 | 34 | 74 | 33 |
| **7** | 40 | 35 | 75 | 59 |

**Suppl. Table 2.** Bacterial Reads of Moraxella catarrhalis and Rothia mucilaginosa in tumor and normal samples

| **case** | **type** | **bacteria** | **absolute count** | **relative (in %)** |
| --- | --- | --- | --- | --- |
| Case 1 | normal | Moraxella catarrhalis | 0 | 0 |
| Case 1 | normal | Rothia mucilaginosa | 51 | 0.0129 |
| Case 1 | tumor | Moraxella catarrhalis | 120 | 0.0005 |
| Case 1 | tumor | Rothia mucilaginosa | 2104 | 0.0081 |
| Case 2 | normal | Moraxella catarrhalis | 0 | 0 |
| Case 2 | normal | Rothia mucilaginosa | 23 | 0.0017 |
| Case 2 | tumor | Moraxella catarrhalis | 28 | 0.0006 |
| Case 2 | tumor | Rothia mucilaginosa | 663 | 0.015 |
| Case 3 | normal | Moraxella catarrhalis | 17 | 0.0014 |
| Case 3 | normal | Rothia mucilaginosa | 59 | 0.005 |
| Case 3 | tumor | Moraxella catarrhalis | 14 | 0.0002 |
| Case 3 | tumor | Rothia mucilaginosa | 1835 | 0.028 |
| Case 4 | normal | Moraxella catarrhalis | 23 | 0.0004 |
| Case 4 | normal | Rothia mucilaginosa | 1924 | 0.0372 |
| Case 4 | tumor | Moraxella catarrhalis | 15 | 0.0001 |
| Case 4 | tumor | Rothia mucilaginosa | 1677 | 0.008 |
| Case 5 | normal | Moraxella catarrhalis | 4 | 0.0004 |
| Case 5 | normal | Rothia mucilaginosa | 127 | 0.0119 |
| Case 5 | tumor | Moraxella catarrhalis | 6 | 0.0002 |
| Case 5 | tumor | Rothia mucilaginosa | 2380 | 0.0597 |
| Case 6 | normal | Moraxella catarrhalis | 46 | 0.0029 |
| Case 6 | normal | Rothia mucilaginosa | 333 | 0.021 |
| Case 6 | tumor | Moraxella catarrhalis | 31 | 0.0006 |
| Case 6 | tumor | Rothia mucilaginosa | 478 | 0.009 |
| Case 7 | normal | Moraxella catarrhalis | 2 | 0.0001 |
| Case 7 | normal | Rothia mucilaginosa | 435 | 0.0239 |
| Case 7 | tumor | Moraxella catarrhalis | 26 | 0.0003 |
| Case 7 | tumor | Rothia mucilaginosa | 1048 | 0.012 |
